# Advice-Taking for Objective Face Age Estimates Relative to Subjective Face Trustworthiness Estimates

**DOI:** 10.3390/bs15060809

**Published:** 2025-06-13

**Authors:** Joseph R. Phillips, Gabrielle Weidemann, Natalie C. Ebner, Phoebe E. Bailey

**Affiliations:** 1 Faculty of Health, University of Technology Sydney, Sydney, NSW 2007, Australia; joseph.phillips@uts.edu.au; 2 School of Psychology, Western Sydney University, Sydney, NSW 2751, Australia; g.weidemann@westernsydney.edu.au; 3 MARCS Institute for Brain, Behaviour, and Development, Western Sydney University, Sydney, NSW 2751, Australia; 4 Department of Psychology, University of Florida, Gainesville, FL 33126, USA; natalie.ebner@ufl.edu

**Keywords:** advice-taking, weight of advice, subjective estimate, objective estimate, trustworthiness

## Abstract

This study examined whether advice-taking differs when making subjective face trustworthiness estimates relative to objective face age estimates. Participants (*N* = 177) completed a judge–advisor system task to measure how much weight they would give to advice regarding the age of a face (i.e., an objective estimate with a single correct answer) versus the trustworthiness of a face (i.e., a subjective estimate without a correct answer). Measures of fluid intelligence and working memory assessed cognitive resources, and participants provided ratings of perceived difficulty and confidence for each type of estimate. The difference between initial estimates and advice provided a measure of actual difficulty for each type of estimate. We found that advice-taking was greater when estimating face age than face trustworthiness. In addition, perceived difficulty, confidence, and actual difficulty were greater for face trustworthiness than age estimates. However, greater advice-taking was associated with greater actual difficulty for face age estimates, and less actual difficulty for face trustworthiness estimates. While previous research suggests that advice-taking increases with task difficulty, the current data reveal that this may depend on type of estimate. Subjective trustworthiness estimates that were more difficult to estimate than objective age estimates were associated with less advice-taking, and this may be at least partly attributable to differing motivations underlying objective (age) vs. subjective (trustworthiness) estimations.

## 1. Introduction

We often consider advice from others when making estimations. While advice-taking research using the judge–advisor paradigm has focused on objective, fact-based estimations (e.g., number of coins in a jar or distance between two cities), few studies have examined estimations that are based on subjective opinion (e.g., quality of a restaurant or doctor, or likeability of a movie or holiday destination) (Bailey et al., 2023). This is despite subjective estimates being ubiquitous in everyday life, in both face-to-face and online settings. Moreover, there are grounds for predicting that advice-taking may differ when making an objective versus a subjective estimate based on different underlying motivations. Indeed, a recent meta-analysis of 129 independent datasets revealed that more weight is given to advice when an estimate is subjective relative to objective (Bailey et al., 2023). However, it is important to note that most studies included in the meta-analysis assessed objective estimates, and most assessed either objective or subjective estimate advice-taking and did not directly compare the two in a within-subjects design. The current study aims to directly compare objective vs. subjective estimate advice-taking within a single sample to contribute to an understanding of the degree to which subjective judgments might be influenced by advice. A further aim is to better understand the different mechanisms potentially underpinning the incorporation of advice into objective versus subjective estimations.

Objective (or intellective; Laughlin, 1980) estimates are based on verifiable data or facts, with one correct answer, while subjective (or judgement; Laughlin, 1980) estimates are based on personal opinions or beliefs, where there is no single correct answer. Advice-taking in relation to an objective estimate offers the opportunity to improve accuracy. Indeed, the combined estimates of a group tend to be considered more accurate than an estimate from an individual (Mannes, 2009). In contrast, taking advice when making a subjective estimate may suggest that advice-taking is not always dominated by accuracy-seeking informational motives (See et al., 2011; Van Swol, 2011) but may also assess normative social influence and the motivation to maintain social harmony and to align with others to achieve consensus (Laughlin & Ellis, 1986; Mahmoodi et al., 2015). Taking advice when making a subjective estimate may also suggest that the judge perceives their own knowledge of the estimate to be uncertain due to the absence of a “right or wrong” estimate, and this uncertainty may increase advice-taking (Yaniv, 2004; Yaniv & Kleinberger, 2000).

According to Bonaccio and Dalal (2006), uncertainty reduces a judge’s confidence, which in turn contributes to increased advice-taking. Indeed, a judge’s confidence in their own estimation has an inverse effect on how much weight they give to advice (See et al., 2011). While a subjective estimate may evoke uncertainty, which reduces confidence, both objective and subjective estimates may be associated with reduced confidence to the extent that the estimation task is perceived to be more difficult. Indeed, Wang and Du (2018) observed greater advice-taking when they made a perception task harder by blurring the stimuli, demonstrating that increased difficulty increased the weight of advice. Our study aimed to directly compare the influence of both perceived difficulty and decision-maker confidence on advice-taking in relation to an objective versus a subjective estimate. Given that greater fluid intelligence and poorer working memory have previously correlated with greater rated value of expert and novice advice, respectively (Bailey et al., 2021), we also examined correlations between these measures and advice-taking.

We investigated advice-taking behaviour using the judge–advisor system paradigm (Meshi et al., 2012; Sniezek & Buckley, 1995). In this paradigm, participants are required to give an estimate, view advice from an outside source, and then provide a new estimate. The judge–advisor system paradigm allows for a precise index of advice-taking on a scale from 0 (completely disregarded advice) to 1 (relied completely on advice). The optimum weight of advice in this paradigm is suggested to be 0.5 (i.e., an equal weight placed on the judge’s estimate and the advice; Larrick & Soll, 2006). Interestingly, however, individuals tend to engage in egocentric discounting, which refers to the inflation of their own judgement relative to the advice they receive (Yaniv & Kleinberger, 2000). However, the perceived quality of the advice or advisor typically affects advice-taking, with greater weight given to higher quality advice (Bailey et al., 2023). The current study removed any information about the advisor to ensure advice-taking was neither at ceiling nor floor to increase the likelihood of detecting an effect of subjective vs. objective estimates on weight of advice. To further control for potentially confounding variables, we aligned the two estimation tasks by using the same stimuli for each type of task. Participants were shown images of faces and were asked to estimate the age of those faces (i.e., the objective estimate) and the trustworthiness of the faces (i.e., the subjective estimate). Faces provide a superficial indication of trustworthiness, which is a subjective judgement based on external cues such as face shape, expression, and attractiveness. Inter-rater agreement on ratings of trustworthiness from face stimuli is generally moderate to high but lower than ratings of perceived age (Kramer et al., 2018), and there is no correct response when judging facial trustworthiness based on appearance. Nevertheless, many real-world judgements are made based on both perceived age and perceived trustworthiness.

The overall aim of the current study was to investigate whether estimating face age vs. face trustworthiness would influence how much weight was given to advice. In line with our meta-analysis (Bailey et al., 2023), the first hypothesis was that participants would give more weight to advice when making a subjective estimate about the trustworthiness of a face relative to an objective estimate about the age of a face. The second hypothesis was that greater perceived difficulty of an estimate would be correlated with lower judge confidence and greater advice-taking. To further examine this hypothesis, we tested whether greater task difficulty was associated with lower fluid intelligence and working memory scores. We also explored whether confidence, perceived difficulty, and actual difficulty would differ for objective face age vs. subjective face trustworthiness estimates.

## 2. Methods

### 2.1. Participants

One hundred and seventy-seven participants (49% female) were recruited using Prolific (www.prolific.com) and were reimbursed AUD 5 for 15 min of their time. Participants were aged 18 to 82 years (*M* = 35.50, *SD* = 12.50) and resided in Australia. All participants provided informed consent, and the study was approved by the Human Research Ethics Committee at the University of Technology Sydney (ETH22-6857). All participants accurately responded to two attention check questions. According to G*Power version 3.1.9.7, 158 participants are required to detect a small-to-medium effect (*f* = 0.13; alpha = 0.05) with 90% power and two repeated measurements.

### 2.2. Measures and Procedure

#### 2.2.1. Judge–Advisor Task

The weight of advice was measured using the judge–advisor system paradigm (Sniezek & Buckley, 1995). In the current study, participants completed two blocks of 24 trials. Each block presented the same 24 faces in random order. One block required participants to estimate the age of each face while the other block required participants to estimate the trustworthiness of each face. The order of the age and trustworthiness blocks was randomised across participants.

Each face trustworthiness estimate block would start with the text “you will judge how trustworthy you think each face is using a scale from 0 (not at all trustworthy) to 100 (completely trustworthy). After each estimate you will receive advice. You will then have an opportunity to revise your estimate.” Participants were then presented with a face and prompted to provide a trustworthiness estimate. Once they entered their estimate, the picture would remain while they were given advice without any information regarding the source of the advice, “Your estimate for this person was <estimate> out of 100; The advice is <advice> out of 100; You can now provide a revised estimate of the trustworthiness of this face”. Note that “<estimate>” was replaced with the first estimate given by the participant, and “<advice>” was replaced with the average trustworthiness estimate of 199 participants in a previous study (Pehlivanoglu et al., 2023).

Face age estimate blocks were structured in an identical manner, and each block started with brief instructions, “you will be required to estimate the age of some faces. After each estimate you will receive advice. You will then have an opportunity to revise your estimate.” A face was then presented, and participants were prompted to estimate the age between 18 and 80 years. The picture would remain while a second prompt appeared, “You estimated the age as <estimate>, the advice is <advice>. You can now provide a revised estimate of the age”. The advice was the average age estimated by 154 participants in a previous study (Ebner et al., 2010).

Face images of young (*M* = 26.7 years, *SD* = 3.5), middle-aged (*M* = 47.0 years, *SD* = 3.85), and older (*M* = 68.6 years, *SD* = 2.3) males and females were taken from the FACES database (Ebner et al., 2010). We selected four young male, four young female, four middle-aged male, four middle-aged female, four older male, and four older female faces. Within each subgroup of four faces, two of the faces represented the least trustworthy and two faces the most trustworthy based on the Pehlivanoglu et al. (2023) dataset to provide high (*M* = 58.5, *SD* = 6.5) and low (*M* = 46.2, *SD* = 6.0) trustworthy faces within each subgroup on a scale from 0 (not at all trustworthy) to 100 (extremely trustworthy). To assess reliability of initial estimates we calculated Kendall’s W using the seolmatrix module in jamovi (version 2.6.26). This revealed slight-to-fair agreement for subjective trustworthiness ratings (W = 0.20, *p* < 0.001) and substantial agreement for the objective age ratings (W = 0.77, *p* < 0.001). Note that values of more than 3 *SD*s from the type of estimate mean were removed prior to the reliability analysis (i.e., 1.2% of all initial estimates).

Our outcome variable *weight of advice* was calculated as [(final estimate − initial estimate)/(advice − initial estimate)]. According to this calculation, 0 indicates no influence of advice (i.e., the initial and second estimates are the same), while 1 indicates full reliance on the advice (i.e., the second estimate is equal to the advised estimate).

#### 2.2.2. Confidence and Perceived Task Difficulty

After completing both the age and trustworthiness estimation blocks, participants were asked to rate how difficult each estimation task was overall across all trials, as well as how confident they were in their initial estimates across all trials. These ratings were provided once both blocks of estimation tasks had been completed, using a 7-point Likert scale ranging from 1 (not at all) to 7 (very difficult/confident).

#### 2.2.3. Actual Task Difficulty

A second, more objective measure of task difficulty was created for each type of estimate. We subtracted the judge’s initial estimate on each trial from the advice on that trial, noting that the advice on each trial was the average estimate provided by groups of participants in Ebner et al. (2010) and Pehlivanoglu et al. (2023) for face age and face trustworthiness, respectively. Because age was estimated on an 18–80 scale (63 scale points) and trustworthiness on a 0–100 scale (101 scale points), the face age and face trustworthiness difference scores were standardised to control for the differing scales. Age differences were divided by 63, and trustworthiness differences were divided by 101. The higher the difference score, the more objectively difficult the estimation task.

#### 2.2.4. Cognitive Capacity

Raven’s Matrices (Raven et al., 1998) were used to measure fluid intelligence. Due to time restrictions, 15 items were selected from sets C, D, and E from the original Raven’s Progressive Matrices sets. Two of these items were given as practice, with clear instructions and reasoning to support the correct answer. This approach resulted in the highest possible score being 13. Participants had 60 s to complete each item, as this has been demonstrated to provide the minimum time without compromising the validity of each item (Tatel et al., 2022).

The Backward Digit Span from the WAIS-R (Wechsler, 2008) was used to measure working memory. This test consisted of sets of digits containing 1 to 9 numbers, with the sets gradually increasing length. For each set, participants were presented one number for 1 s, in a sequential order. At the end of each set, participants were prompted to type the numbers in the reverse order of presentation. Two sets for each length were presented, with participants only required to enter the correct digits for one of the sets to advance to the next set length. If participants entered the incorrect number sequence for both sets, the test stopped with the current set length recorded as their backward digit span. The highest possible score on this measure was 7.

### 2.3. Judge–Advisor Task Data Cleaning

In line with Bailey et al. (2021), three data cleaning processes were undertaken. First, all trials where the participant’s first estimate matched the advice, and therefore could not provide a measure of advice-taking, were removed. This approach resulted in exclusion of 2.6% of the face age trials and 1.0% of the face trustworthiness trials. Second, trials with weights of advice less than −1.3 or greater than 1.3 were also removed because such weights have been shown to incorrectly suggest that participants were greatly influenced by advice (see Bailey et al., 2021; Meshi et al., 2012; Schultze et al., 2017). This excluded 0.6% of the face age trials and 1.4% of the face trustworthiness trials. Third, all trials with a weight of advice greater than 3 *SD*s above or below the mean were adjusted to 3 *SD*s above or below the mean, respectively, to reduce the influence of outlier data points. This resulted in the adjustment of 0.08% of the face age trials and 0.07% of the face trustworthiness trials.

### 2.4. Data Analysis

A repeated measures Analysis of Variance (ANOVA) examined the first hypothesis regarding how the type of estimate (age, trustworthiness) affected weight of advice. Next, we calculated correlations to examine the second hypothesis relating to associations between confidence, task difficulty, and weight of advice, as well as correlations between cognitive measures and task difficulty. Spearman’s correlations were calculated since the data consisted of both ordinal and continuous measures. Due to the ordinal scales, we used Wilcoxon’s signed rank tests to compare ratings of confidence and perceived difficulty for each type of task, and a *t*-test was used to compare the continuous measures of actual task difficulty for each type of estimate.

## 3. Results

### 3.1. Weight of Advice

**Figure 1:**
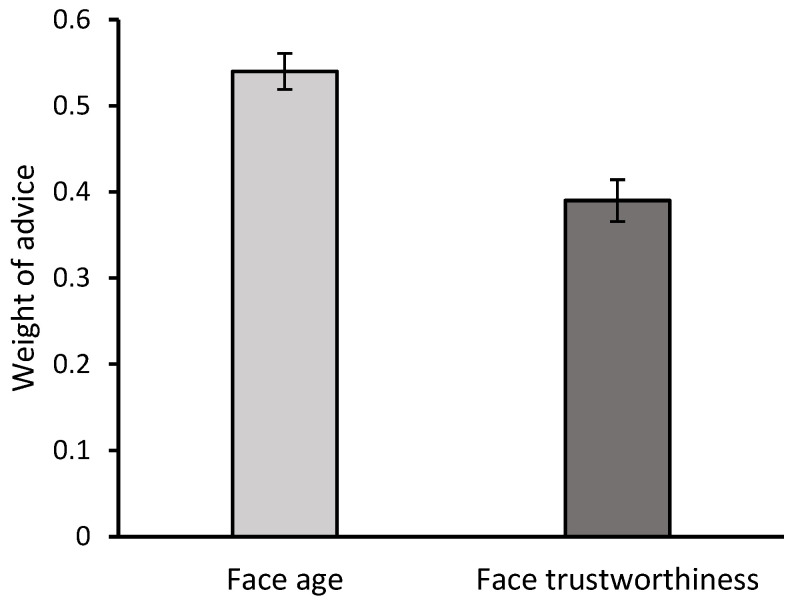
Weight of advice when estimating face age and face trustworthiness. Error bars represent the standard error of the mean.

The ANOVA revealed a significant effect of estimate type, *F*(1, 177) = 56.2, *p* < 0.001, η^2^*_p_* = 0.24, with greater weight given to advice regarding the face age estimate (*M* = 0.54; *SD* = 0.28) compared to the face trustworthiness estimate (*M* = 0.39; *SD* = 0.32) (see Figure 1). The effect remained significant after controlling for block order (age vs. trustworthiness), *F*(1, 175) = 20.74, *p* < 0.001, η^2^*_p_* = 0.11.

### 3.2. Correlations

As shown in Table 1, greater weight of advice was correlated with greater perceived difficulty for both types of estimation. In addition, greater perceived difficulty was correlated with lesser confidence for both types of estimation. Greater weight of advice was correlated with greater actual difficulty for face age estimates and with less actual difficulty for the face trustworthiness estimates. Lower fluid intelligence scores were associated with greater weight of advice for the face trustworthiness estimates, while lower working memory scores were associated with greater actual difficulty when estimating face trustworthiness.

### 3.3. Perceived Difficulty and Confidence

Participants perceived face trustworthiness estimations (*M* = 6.00, *SD* = 1.58) to be more difficult than face age estimations (*M* = 5.02, *SD* = 1.51; *W* = 4798, *p* = 0.004, *r_rb_* = 0.300). However, participants were also more confident in their estimates for face trustworthiness (*M* = 6.00, *SD* = 1.42) than face age (*M* = 6.00, *SD* = 1.37; *W* = 4598, *p* = 0.006, *r_rb_* = 0.288).

### 3.4. Actual Difficulty

The average standardised face age estimate difference (*M* = 10.8%; *SD* = 5.92) was significantly lower than the average standardised face trustworthiness estimate difference (*M* = 13.0%; *SD* = 5.69; *t*(176) = 3.92, *p* < 0.001, *d* = 0.40). That is, actual difficulty was greater for face trustworthiness estimates than face age estimates.

## 4. Discussion

The current study aimed to investigate the degree to which advice is incorporated into judgements about face trustworthiness (a subjective estimate) versus face age (an objective estimate). We examined how the judge’s confidence and perceived task difficulty influenced advice-taking for each type of estimate. In direct contrast to our first hypothesis, greater weight was given to advice for objective (i.e., face age) relative to subjective (i.e., face trustworthiness) estimates. However, our second hypothesis was supported in that greater perceived estimation difficulty was correlated with both lower confidence and greater advice-taking. These associations were evident for both subjective and objective estimates. In addition, the judge’s confidence, perception of task difficulty, and actual difficulty were lesser for face age than face trustworthiness estimates. Interestingly, however, using the objective measure of actual task difficulty, greater advice-taking was associated with greater difficulty for objective face age estimates but less difficulty for subjective face trustworthiness estimates.

Greater advice-taking for an objective relative to a subjective estimate is inconsistent with the finding in Bailey et al.’s (2023) meta-analysis. However, only two previous studies to the best of our knowledge have directly compared objective and subjective estimate advice-taking within a single sample (i.e., Van Swol, 2011, Experiments 1 and 2). Consistent with the current data, Van Swol’s Experiment 1 demonstrated greater advice-taking for an objective estimate (math problems) relative to a subjective estimate (movie preference ratings). Although difficulty was not directly measured, Van Swol suggested that the difference in advice-taking was potentially attributable to the maths estimates being more difficult than the movie preference estimates. It was proposed that people may have relied more on advice to reduce their cognitive load. Consistent with this idea, and previous research (e.g., Hoffmann et al., 2020), greater perceived difficulty when making an estimate in the current study was associated with a greater degree of advice-taking. However, this was evident for both objective and subjective estimates, and participants perceived the subjective estimates (face trustworthiness) to be more difficult than the objective estimates (face age). In addition, lower working memory and fluid intelligence scores were associated with greater actual difficulty and greater advice-taking, respectively, when estimating face trustworthiness, but not face age. This provides further evidence to suggest that face trustworthiness was more difficult to estimate than face age. Indeed, actual task difficulty was also greater when estimating face trustworthiness than age.

Previous research has shown that greater confidence is associated with reduced advice-taking (Bonaccio & Dalal, 2006; See et al., 2011). While confidence ratings did not correlate with weight of advice for either type of estimate in the current study, participants rated confidence higher for face trustworthiness than face age estimates and were less willing to take advice based on face trustworthiness than face age estimates. Greater confidence in initial estimates was also associated with lower ratings of perceived difficulty for both types of estimation tasks. Taken together, confidence may not provide an explanation for the greater weight of advice when estimating face age relative to face trustworthiness. A potential limitation of the current study was that the rating of confidence and perceived difficulty took place after the completion of all judge–advisor trials. Future studies should take these ratings immediately before and after each trial to determine whether the timing of the ratings influenced the associations between ratings of confidence and advice-taking. Our objective measure of actual task difficulty was arguably a more reliable measure than confidence or perceived difficulty because it was calculated based on the difference between initial estimates and advice on every trial. Moreover, a novel finding in the current study was that greater task difficulty, as assessed objectively, was associated with greater advice-taking for the objective face estimate but less advice-taking for the subjective face trustworthiness estimate.

When participants’ initial face age estimates were further from the advice (i.e., more objectively difficult), they took more advice. In contrast, when their initial face trustworthiness estimates were closer to the advice (i.e., less objectively difficult), they took more advice. It should be noted that the advice in the current study was the average estimate provided by groups of participants in Ebner et al. (2010) and Pehlivanoglu et al. (2023) and thus provided good estimations of face age, as well as good indicators of consensus in relation to face trustworthiness. As noted, relative to objective estimates that entail accuracy-seeking informational motivations, advice-taking for subjective estimates with no single ‘correct’ answer may represent normative social influence and the motivation to align with others’ opinions to reach consensus (Mahmoodi et al., 2015). Similarly, there is an understanding that subjective values are typically determined by the aggregation of multiple estimates (Laughlin & Ellis, 1986). These different underlying social vs. informational motivations may help to explain the current data.

It is possible that the judge is more trusting of advice that more closely aligns with their initial judgement (Van Swol, 2011), and this was demonstrated for subjective, but not objective, estimates in the current study. When informational goals are dominant, an initial objective estimate (age) that aligns more closely with an advised estimate may be perceived to be more accurate, which in turn increases egocentric discounting of advice. When social goals are dominant, an initial subjective estimate (trustworthiness) that is closer to the advised estimate may increase advice-taking because the judge is seeking support for their own initial decision (Sherman et al., 1984). This proposition is consistent with a theory of bias amplification, which describes how perceiving social information to support one’s own biased belief can amplify that belief (Hardy et al., 2023). Indeed, Allan et al. (2025) demonstrated a concerning amplification of baseline biases by biased generative AI output. The current data extend this effect to biased facial stereotyping, which is consistent with Jaeger et al.’s (2020) finding that providing facial trustworthiness information that aligns with an initial verdict increases facial bias in jury verdicts.

A further possibility is that participants explicitly recognised that the subjective estimate was subjective, and therefore taking advice was immaterial. This could be examined in future studies involving subjective and objective estimates matched for difficulty. It also might be argued that the current study did not elicit sufficient motivation to reach consensus because effects of advice quality were controlled for by removing any information about the advice and advisor. Alternatively, perceived advice quality may be influenced by the perception of age-related advice as objective and trustworthiness-related advice as subjective. To test these ideas, future research should examine whether advice from an individual versus a group differentially influences advice-taking when making objective versus subjective estimates. Future studies should also measure perceived quality of advice and should specifically examine whether face trustworthiness estimates rely more on consensus than other types of subjective estimates such as ratings of the competence of a health professional or the likability of a movie. A further potential limitation of the current study was the inclusion of only one type of objective estimate and only one type of subjective estimate. Future studies of advice-taking should include more than one type of subjective and objective estimate and systematically vary the extent to which those estimates rely on cognitive resources and/or a need for consensus with others.

In conclusion, advice relating to objective face age estimations that are based on fact was given more weight than advice relating to subjective face trustworthiness estimations that are based on opinion. While greater reliance on advice was associated with greater perceived difficulty for both subjective and objective estimations, face trustworthiness was perceived to be more difficult to estimate than face age and was indeed more difficult as assessed objectively. Moreover, greater task difficulty, as measured objectively, was associated with greater advice-taking for face age estimates but less advice-taking for face trustworthiness estimates. Studies using the judge–advisor paradigm have typically examined objective estimates for which advice-taking appears to be driven by informational goals, the pursuit of accuracy, and egocentric discounting (Bailey et al., 2021). The current study demonstrates that incorporating advice into a subjective estimate such as face trustworthiness may be underpinned by social processes, such as desire to conform with a group, and has the potential to amplify biases based on stereotypical beliefs.

## Figures and Tables

**Table 1:** Spearman’s correlations between cognitive measures, and weight of advice, difficulty and confidence for each type of estimate.

	1	2	3	4	5	6	7	8	9	10
1. Working memory	-									
2. Fluid intelligence	0.294 ***	-								
**Age**										
3. Weight of advice	0.041	−0.116	-							
4. Perceived difficulty	0.082	0.007	0.159 *	-						
5. Actual difficulty	−0.063	−0.104	0.369 ***	0.306 ***	-					
6. Confidence	0.095	−0.055	−0.061	−0.257 ***	−0.029	-				
**Trustworthiness**										
7. Weight of advice	−0.015	−0.201 **	0.596 ***	0.058	0.116	−0.062	-			
8. Perceived difficulty	−0.009	0.065	0.184 *	0.300 ***	0.001	−0.362 ***	0.190 *	-		
9. Actual difficulty	−0.225 *	−0.003	−0.006	0.019	0.102	0.010	−0.162 *	−0.019	-	
10. Confidence	−0.064	−0.062	−0.114	−0.428 ***	−0.290 ***	0.327 ***	−0.028	−0.201 **	0.049	-

Note. * *p* < 0.05; ** *p* < 0.01, *** *p* < 0.001.

## Data Availability

The datasets generated during and/or analysed in the current study are available in OSF (https://osf.io/8gvkf/files/osfstorage/66415906419d005a4afe9cf).
